# Disproportionate Cardiovascular Risk in Women with Type 2 Diabetes: A Narrative Review of Diet, Metabolic Phenotypes, and Gene–Diet–Epigenetic Interactions Across the Life Course

**DOI:** 10.3390/nu18081217

**Published:** 2026-04-12

**Authors:** Tatjana Ábel, Diána Gellért, Éva Csobod Csajbókné, Erzsébet Mák

**Affiliations:** Department of Dietetics and Nutritional Sciences, Faculty of Health Sciences, Semmelweis University, 1088 Budapest, Hungarycsajbokne.csobod.eva@semmelweis.hu (É.C.C.); mak.erzsebet@semmelweis.hu (E.M.)

**Keywords:** type 2 diabetes, cardiovascular disease, sex differences, women with type 2 diabetes, dietary patterns, nutrigenetics, epigenetics, menopause, pregnancy, insulin resistance, endothelial dysfunction, microvascular dysfunction, precision nutrition

## Abstract

**Background**: Cardiovascular disease (CVD) remains the leading cause of morbidity and mortality among individuals with type 2 diabetes mellitus (T2DM). Although women generally exhibit a more favorable cardiovascular risk profile than men in the general population, this protection is substantially reduced in the presence of diabetes, resulting in a disproportionately greater relative increase in CVD risk among women. **Objective**: This review aims to integrate the roles of metabolic phenotypes, dietary exposures, and genetic susceptibility in shaping cardiovascular risk in women with T2DM, with a focus on diet–gene and diet–epigenetic interactions across critical stages of the female life course. **Methods**: A narrative review of epidemiological, clinical, and mechanistic evidence from recent literature was conducted to synthesize current knowledge on sex-specific cardiometabolic pathways and nutritional determinants of vascular risk in T2DM. **Results**: Current evidence indicates that several interconnected mechanisms contribute to enhanced cardiovascular vulnerability in diabetic women, including (i) adipose tissue dysfunction and ectopic fat accumulation; (ii) insulin resistance with metabolic inflexibility and lipotoxicity; and (iii) endothelial and microvascular dysfunction driven by impaired nitric oxide signaling. Dietary patterns modulate these pathways through effects on inflammation, oxidative stress, postprandial lipid metabolism, and vascular function. Emerging evidence highlights that genetic variants (e.g., APOE; CETP; TCF7L2) significantly modify metabolic responses to dietary exposures in patients with T2DM; supporting a role for nutrigenetic interactions in shaping cardiovascular risk. In parallel, diet-related epigenetic mechanisms—including metabolic memory and early-life programming—may contribute to long-term and potentially intergenerational cardiometabolic risk. **Conclusions**: Integrating dietary patterns with genetic susceptibility and epigenetic regulation provides a mechanistic framework for understanding the disproportionate cardiovascular risk in diabetic women and supports the development of sex-specific, life-course-oriented precision nutrition strategies for cardiovascular risk reduction

## 1. Introduction

Cardiovascular disease (CVD) remains the leading cause of morbidity and mortality in type 2 diabetes mellitus (T2DM), with a disproportionately greater relative risk observed in women compared with men [1]. In the general population, women typically exhibit a lower baseline risk of cardiovascular events than men; however, this advantage is markedly attenuated—or even reversed—in the presence of diabetes, creating a clinically relevant and insufficiently explained sex-specific risk paradox [2,3]. Large-scale meta-analyses and real-world cohorts consistently demonstrate that women with T2DM experience a disproportionately higher *relative* risk of coronary heart disease, stroke, and cardiovascular mortality compared with men with diabetes, even after adjustment for conventional cardiovascular risk factors [4,5,6].

The mechanisms underlying this sex-specific excess cardiovascular risk remain incompletely integrated, particularly across metabolic, nutritional, and genetic domains. Importantly, differences in traditional risk factors—including glycemic control, blood pressure, lipid profiles, and overall adiposity—do not fully explain the magnitude of the observed disparity [3,7,8,9]. These findings suggest the contribution of sex-specific biological pathways that amplify vascular vulnerability and accelerate cardiometabolic deterioration in women with diabetes.

Diet and nutrition represent central and potentially modifiable drivers of cardiometabolic risk in T2DM, interacting with metabolic and vascular pathways that are particularly relevant in women. Dietary patterns and macronutrient composition exert profound effects on glucose homeostasis, lipid metabolism, systemic inflammation, oxidative stress, and endothelial function—pathways that converge to drive atherosclerosis progression and adverse cardiovascular outcomes [9,10,11,12]. Importantly, emerging evidence indicates that dietary quality, adherence to cardioprotective dietary patterns, and eating behaviors may differ between women and men with diabetes, shaped by a complex interplay of biological, hormonal, psychosocial, and socioeconomic factors [13,14]. These sex-related differences in nutritional exposures may contribute to divergent metabolic trajectories and partially underlie the excess cardiovascular risk observed in women with T2DM. Beyond direct metabolic effects, dietary factors interact with genetic susceptibility and epigenetic regulation, forming a key mechanistic axis underlying inter-individual and sex-specific variability in cardiometabolic risk. Advances in nutrigenetics and nutrigenomics suggest that genetic variants influencing insulin signaling, lipid metabolism, and inflammatory pathways may modify cardiometabolic responses to dietary exposures. These interactions may partly explain inter-individual variability in metabolic risk among individuals with T2DM. Conversely, long-term dietary patterns may induce epigenetic modifications that influence vascular and metabolic risk across the life course [15,16,17]. In women, these interactions are further amplified across hormonally sensitive life-course transitions—particularly pregnancy and menopause—representing critical windows of cardiometabolic vulnerability [18,19,20].

To our knowledge, no integrative framework has comprehensively linked sex-specific cardiometabolic phenotypes with dietary exposures, genetic susceptibility, and epigenetic programming across the female life course in T2DM. While previous reviews have addressed individual components of this relationship, including sex differences in cardiovascular risk, dietary determinants, and genetic or epigenetic mechanisms, these domains have largely been investigated in isolation [2,3,5,6,9,10,11,12]. Current risk stratification frameworks and preventive strategies are still largely extrapolated from male-dominated cohorts, and may therefore inadequately capture sex-specific mechanisms and critical windows of risk across the female life course.

In this context, a deeper understanding of how diet interacts with genetic predisposition, epigenetic regulation, and hormonally mediated metabolic transitions to shape cardiovascular health in women with T2DM is urgently needed. Such an integrative perspective is particularly relevant given the modifiable nature of nutritional exposures and the potential of personalized, sex-aware dietary interventions to reduce long-term cardiovascular risk.

This review aims to integrate current evidence on sex-specific cardiometabolic phenotypes, dietary determinants, and gene–diet–epigenetic interactions to explain the excess cardiovascular risk in women with T2DM. In addition, we examine the role of specific dietary patterns and pro-inflammatory nutritional exposures, and discuss their mechanistic links to metabolic and vascular dysfunction. Finally, we address the clinical implications of these findings and highlight key knowledge gaps to inform future research and the development of sex-specific, nutrition-based prevention strategies in diabetes.

## 2. Literature Search Strategy

We conducted this narrative review using a structured and reproducible literature search method in order to provide a comprehensive and conceptually balanced overview of gender-specific cardiometabolic risk in women with type 2 diabetes, as well as relevant evidence regarding diet–gene–epigenetic interactions.

We conducted electronic database searches in PubMed, Web of Science, and ClinicalTrials.gov. The primary search period covered studies published between January 2000 and December 2025, supplemented as needed with earlier landmark studies.

Search strategies combined controlled vocabulary and free-text keywords related to the following areas: (i) type 2 diabetes mellitus, (ii) cardiovascular diseases and vascular dysfunction, (iii) sex differences and woman-specific factors, (iv) dietary habits and nutritional exposures, and (v) nutritional genetics and epigenetic mechanisms. Representative search terms included the following combinations: “type 2 diabetes,” “cardiovascular diseases,” “women,” “gender differences,” “diet,” “eating habits,” “nutrigenetics,” “gene–diet interaction,” “epigenetics,” “DNA methylation,” “inflammation,” and “endothelial dysfunction.”

To ensure conceptual rigor, we surveyed the literature and selected studies based on predefined thematic areas that reflect the central framework of this review: (i) non-specific cardiometabolic phenotypes, (ii) dietary exposures and nutritional habits, and (iii) gene–diet and epigenetic interactions throughout women’s lifetimes. We employed this domain-based approach to ensure a balanced presentation of epidemiological, clinical, and mechanistic evidence and to minimize selective reporting.

Studies were eligible if they met the following criteria: (i) original research articles, clinical trials, cohort studies, systematic reviews, or meta-analyses; (ii) they examined cardiometabolic or cardiovascular outcomes of type 2 diabetes; (iii) they included data on dietary exposures, metabolic phenotypes, or gene–diet/epigenetic interactions; and (iv) they were published in English. We considered both human and mechanistic studies, provided that the experimental results were clearly relevant to human cardiometabolic physiology.

Studies were excluded if: (i) they were not relevant to the scope of this review; (ii) they were conducted exclusively in animal models without a translational context; or (iii) they did not address cardiometabolic, nutritional, genetic, or epigenetic aspects related to type 2 diabetes.

Study selection was performed through a stepwise screening of titles, abstracts, and full texts based on their relevance to predefined thematic areas. Due to the narrative structure, we did not apply formal systematic review procedures (e.g., PRISMA-based selection or quantitative quality scoring); however, we incorporated elements of structured evidence identification and thematic categorization to enhance transparency and methodological rigor.

We employed a narrative synthesis approach, ranking studies based on methodological quality, consistency of results, and relevance to non-specific cardiometabolic mechanisms. Where available, we gave preference to evidence from large-scale meta-analyses, randomized controlled trials, and well-characterized cohort studies. We placed particular emphasis on studies presenting gender-specific or gender-stratified results; however, due to the limited availability of such data, we also incorporated mechanistic and mixed-gender evidence to support biological plausibility.

Potential sources of bias include the narrative nature of the review, the heterogeneity of study designs, and the limited availability of gender-disaggregated data. We addressed these limitations by applying a predefined conceptual framework, ensuring comprehensive coverage of key areas, and prioritizing high-quality, mechanistically coherent evidence.

## 3. Sex Differences in Cardiovascular Risk in Type 2 Diabetes: Implications for Women

Understanding sex differences in cardiovascular risk and traditional risk factor profiles provides an essential clinical and epidemiological framework for interpreting the downstream biological mechanisms discussed in this review. These differences not only define the baseline risk landscape in women with T2DM but also influence how dietary exposures, genetic susceptibility, and epigenetic regulation interact to shape cardiometabolic outcomes.

### 3.1. Epidemiology of Cardiovascular Risk in T2DM: Why Sex Matters

Although the excess cardiovascular risk associated with T2DM in women is well established, key epidemiological questions remain regarding when this disparity emerges and why it persists in contemporary clinical practice.

Women with T2DM often exhibit a less favorable cardiometabolic profile at the time of diagnosis compared with men, including higher levels of adiposity and a greater clustering of metabolic risk factors, suggesting that women experience a longer subclinical phase before diabetes is recognized, resulting in more advanced metabolic and vascular impairment at diagnosis [9]. Women appear to require a greater degree of metabolic deterioration—particularly in adiposity and insulin resistance—before reaching diagnostic thresholds for T2DM, potentially resulting in more advanced vascular injury by the time treatment is initiated [3,9].

Beyond baseline metabolic burden, real-world studies indicate that women with T2DM are less likely to achieve recommended targets for cardiovascular risk factors, including lipid and blood pressure control, despite guideline-based preventive strategies being widely available [4]. Such differences in the intensity, timing, and effectiveness of preventive care may contribute to residual risk and reinforce the long-term disparity in cardiovascular outcomes between sexes [4].

Collectively, these epidemiological patterns indicate that sex differences in cardiovascular outcomes reflect both biological susceptibility and healthcare-related factors, including delayed diagnosis and suboptimal risk factor control, and persistent gaps in prevention and treatment optimization [4,9]. These considerations provide a key rationale for sex-aware prevention models that emphasize early risk identification and targeted lifestyle strategies, including dietary interventions, as part of a comprehensive approach to reduce cardiovascular burden in women living with T2DM [9].

### 3.2. Traditional Risk Factors Do Not Fully Explain the Sex Gap

Despite a higher burden of conventional cardiovascular risk factors, these differences do not fully account for the observed sex disparity in cardiovascular outcomes [3,8,9,10]. Traditional risk factors—including glycemic control, blood pressure, dyslipidemia, smoking, obesity, and renal dysfunction—remain central drivers of vascular disease progression in diabetes; however, epidemiological analyses consistently demonstrate that the excess *relative* cardiovascular risk in women persists even after adjustment for these variables [2,6,7,8,9,10]. These findings suggest that T2DM may induce a more deleterious cardiometabolic state in women, or that the cumulative duration and severity of risk exposure prior to diagnosis may be greater in women than in men [3,9,10].

Differences in glycemic control have been proposed as a potential explanation, as women with diabetes may experience greater difficulty achieving optimal glycemic targets in real-world settings [4,9,10]. However, hyperglycemia alone does not explain the magnitude of sex-related risk amplification [8,9,10]. Similarly, women with T2DM frequently exhibit adverse lipid profiles and may have less favorable attainment of lipid targets, including lower rates of statin use or lower intensity treatment in some settings, yet adjustment for lipid parameters and lipid-lowering therapy does not eliminate the sex gap, indicating additional mechanisms such as altered lipoprotein functionality and vascular susceptibility [4,8,9,10].

Obesity and adiposity distribution represent particularly relevant factors in women with T2DM. Women are more likely to develop diabetes at a higher body mass index and may accumulate greater levels of adiposity before diagnosis, which may reflect a longer prediabetic period with progressive metabolic deterioration [9,10]. Nevertheless, conventional obesity measures alone are insufficient, highlighting the importance of adipose tissue dysfunction, ectopic fat deposition, inflammation, and endothelial dysfunction, which may contribute additional risk beyond body mass index [9,10,17].

Hypertension is another major determinant of cardiovascular risk in diabetes, and blood pressure control may be suboptimal in women in some real-world cohorts [4,9,10]. Importantly, recent evidence suggests that failure to adequately control conventional cardiovascular risk factors may contribute to worse mortality outcomes in women with T2DM, highlighting the clinical relevance of persistent gaps in risk factor optimization [21]. However, even when blood pressure and lipid variables are accounted for, women remain at disproportionate risk, suggesting that vascular remodeling, arterial stiffness, and microvascular damage may differ by sex in ways not captured by conventional risk metrics, consistent with emerging evidence on sex-specific endothelial and microvascular dysfunction [3,9,10]. Likewise, while smoking prevalence is generally lower in women in many populations, diabetes appears to reduce or eliminate the baseline female advantage in cardiovascular protection, further supporting the role of diabetes-related sex-specific biological vulnerability [2,3,8,9,10].

Taken together, the persistence of excess cardiovascular risk in women with T2DM after adjustment for conventional risk factors indicates that sex-specific mechanisms likely operate beyond standard clinical risk metrics (Table 1) [2,3,8,9,10].

These observations support the need for more refined risk stratification models incorporating sex-aware biomarkers and cardiometabolic phenotyping, and highlight the importance of modifiable pathways—particularly diet-related inflammation, endothelial dysfunction, and metabolic flexibility—in shaping cardiovascular risk in women with T2DM [9,10,11,12,17]. Importantly, these pathways are closely intertwined with genetic susceptibility and epigenetic regulation, providing a mechanistic basis for the gene–diet–epigenetic interactions explored in the following sections.

## 4. Sex-Specific Cardiometabolic Phenotypes Driving Cardiovascular Risk in Women with T2DM

### 4.1. Adipose Tissue Biology and Ectopic Fat Deposition

**Figure 1:**
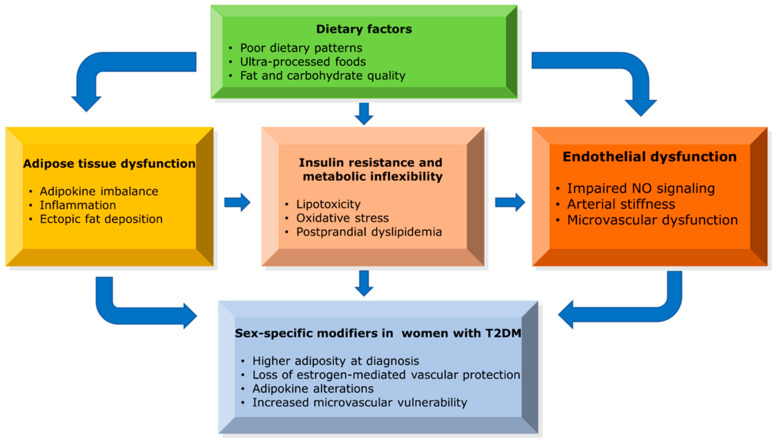
Sex-specific cardiometabolic phenotypes in women with T2DM. Abbreviation: T2DM, type 2 diabetes mellitus.

Adipose tissue dysfunction represents a central mechanistic link between T2DM and CVD, extending beyond the contribution of excess adiposity per se. Qualitative alterations in adipose tissue biology—rather than total fat mass alone—play a critical role in shaping cardiometabolic risk, with important sex-specific implications for women with diabetes [22,23,24]. Impaired adipose tissue expandability—defined as reduced capacity to safely store excess energy—is a key driver of ectopic lipid accumulation and vascular injury (Figure 1) [25,26].

In metabolically healthy states, women preferentially store excess energy in subcutaneous adipose tissue, a pattern associated with preserved insulin sensitivity. However, women develop T2DM at higher levels of adiposity, indicating a prolonged phase of adipose tissue dysfunction before diagnosis [9,27]. Once the functional capacity of subcutaneous adipose tissue is exceeded, lipid spillover toward visceral and ectopic depots ensues, promoting systemic insulin resistance, chronic inflammation, and accelerated atherosclerosis [25,26].

Visceral adipose tissue expansion is a well-established contributor to cardiometabolic risk in T2DM. However, recent imaging and mechanistic studies highlight the particular importance of ectopic fat deposition in amplifying cardiovascular vulnerability, especially in women [24,28,29]. Ectopic lipid accumulation in the liver, skeletal muscle, myocardium, and perivascular adipose tissue induces lipotoxicity, mitochondrial dysfunction, and impaired insulin signaling, thereby exacerbating both metabolic and vascular pathology (Figure 1) [26,30]. Epicardial adipose tissue is a metabolically active depot with local pro-inflammatory and vasocrine effects, and has been associated with coronary artery disease severity and microvascular dysfunction in T2DM [31,32,33].

Sex-specific differences in adipose tissue endocrine and inflammatory profiles may amplify cardiovascular risk in women with diabetes. In clinical studies of women with T2DM, higher circulating leptin and lower adiponectin levels—key adipokines reflecting adipose dysfunction—have been observed and are associated with insulin resistance and CVD risk factors (Figure 1) [9,27]. This unfavorable adipokine milieu promotes endothelial dysfunction, oxidative stress, and vascular inflammation, ultimately impairing nitric oxide bioavailability and accelerating atherogenesis [34,35]. Dysfunctional adipose tissue promotes chronic low-grade inflammation through increased secretion of pro-inflammatory cytokines (e.g., TNF-α, IL-6), contributing to insulin resistance and vascular injury [22,23,36].

Importantly, emerging evidence underscores that adipose tissue dysfunction in women with T2DM is inadequately captured by conventional anthropometric indices (e.g., BMI and waist circumference) [26,28]. Advanced phenotyping studies suggest that qualitative features of adipose tissue—including fibrosis, impaired angiogenesis, reduced adipocyte turnover, and diminished metabolic flexibility—may differ by sex and contribute to divergent cardiometabolic trajectories [25,37,38]. These alterations may predispose women to a metabolic state in which excess caloric intake more readily translates into ectopic lipid deposition and vascular injury, even in the absence of severe obesity.

Dietary composition represents a key modifiable regulator of adipose tissue biology and ectopic fat accumulation, linking nutritional exposures directly to sex-specific cardiometabolic risk pathways. Diets rich in refined carbohydrates, saturated fatty acids, and ultra-processed foods have been shown to exacerbate adipose tissue inflammation, promote macrophage infiltration, and impair adipocyte expandability, thereby facilitating lipid spillover toward ectopic depots [39,40]. In contrast, dietary patterns characterized by higher intakes of unsaturated fatty acids (e.g., olive oil, nuts, fatty fish), dietary fiber, and polyphenol-rich plant foods improve adipose tissue function, enhance adipokine profiles, and reduce visceral and hepatic fat content [11,12,39,40].

Accumulating evidence further suggests that metabolic responses to dietary exposures may differ by sex, positioning adipose tissue as a critical interface through which diet interacts with female-specific biological susceptibility to influence cardiovascular risk in T2DM [14,17].

Collectively, adipose tissue dysfunction and ectopic fat deposition emerge as central mediators of excess cardiovascular risk in women with T2DM. Importantly, these findings support the concept that dietary composition is not merely associated with but actively drives adipose tissue dysfunction and its downstream vascular consequences, highlighting adipose tissue as a key target for nutrition-based, sex-specific cardiovascular prevention strategies.

### 4.2. Insulin Resistance, Metabolic Inflexibility, and Lipotoxicity

Insulin resistance is a central determinant of cardiometabolic and cardiovascular risk in T2DM.

Women tend to develop T2DM at a more advanced stage of metabolic impairment than men, often requiring a greater degree of insulin resistance and adiposity before diagnostic thresholds are reached. As a result, women frequently present with a more adverse metabolic phenotype at the time of T2DM diagnosis, which may contribute to early and accelerated vascular damage [27,41].

In addition to fasting insulin resistance, disturbances in postprandial metabolism play a critical role in shaping cardiovascular risk in T2DM. Impaired postprandial lipid handling, characterized by delayed clearance of triglyceride-rich lipoproteins and prolonged exposure to atherogenic remnant particles, is increasingly recognized as an important driver of endothelial dysfunction and vascular inflammation. In women with T2DM, diabetes attenuates the physiological female advantage in lipid metabolism, making postprandial dyslipidemia a key contributor to cardiovascular risk in women with T2DM [42,43].

Metabolic inflexibility—the reduced capacity to switch between glucose and lipid oxidation in response to nutritional and hormonal signals—represents a key mechanistic link between insulin resistance, ectopic fat accumulation, and lipotoxicity. In T2DM, impaired metabolic flexibility promotes the accumulation of bioactive lipid intermediates, including diacylglycerols and ceramides, in insulin-sensitive tissues such as skeletal muscle, liver, and myocardium. These lipid species interfere with insulin signaling pathways, induce mitochondrial dysfunction, and activate pro-inflammatory cascades, thereby exacerbating metabolic and vascular injury [30,44]. Although metabolic inflexibility is observed in both sexes, differences in sex hormones, adipose tissue distribution, and substrate partitioning may modulate susceptibility to ectopic lipid deposition, potentially amplifying cardiometabolic risk in women once metabolic flexibility is lost [30,41,45].

Diet-induced lipotoxicity represents a central and modifiable pathway linking dietary patterns to insulin resistance and cardiovascular risk, highlighting its relevance for precision nutrition strategies. Diets characterized by excessive energy intake, high-saturated-fat content, refined carbohydrates, and ultra-processed foods promote lipid oversupply to non-adipose tissues, overwhelm oxidative capacity and favor the accumulation of toxic lipid intermediates such as ceramides and diacylglycerols [30,46]. In addition, high-glycemic-load and rapidly absorbable carbohydrate patterns exacerbate postprandial hyperglycemia and insulin demand, thereby reinforcing metabolic inflexibility and worsening lipid handling [42,43]. In contrast, dietary patterns rich in unsaturated fatty acids, dietary fiber, and minimally processed plant-based foods have been shown to improve insulin sensitivity, enhance metabolic flexibility, and reduce ectopic lipid deposition in clinical and experimental studies [46,47,48]. Mediterranean-style and unsaturated-fat-rich dietary approaches may be particularly relevant in this context, as they address both lipid quality and postprandial metabolic stress, two mechanisms closely linked to cardiometabolic deterioration in T2DM [12,47,48].

Given known sex-related differences in lipid metabolism and hormonal regulation, dietary modulation of lipotoxic pathways may hold particular relevance for reducing cardiometabolic and cardiovascular risk in women with T2DM.

Together, insulin resistance, metabolic inflexibility, and diet-induced lipotoxicity form an interconnected pathophysiological triad that contributes to excess cardiovascular risk in women with T2DM (Figure 1). Importantly, these mechanisms are not only biologically interconnected but also nutritionally modifiable, supporting the concept that dietary quality and macronutrient composition should be considered central targets in sex-aware cardiovascular prevention strategies for women with T2DM.

### 4.3. Endothelial Dysfunction, Arterial Stiffness, and Microvascular Disease in Women with T2DM

Endothelial dysfunction (ED) represents an early, integrative vascular phenotype linking hyperglycemia, insulin resistance, dyslipidemia, and chronic inflammation to accelerated atherogenesis and cardiovascular events in T2DM [49,50]. In physiological conditions, premenopausal women without diabetes generally demonstrate more favorable endothelial function and higher nitric oxide (NO) bioavailability than age-matched men; however, diabetes appears to diminish this vascular advantage through converging pathways of oxidative stress, low-grade inflammation, and impaired insulin-mediated endothelial signaling [51]. In T2DM, ED reflects a broader disturbance of vascular homeostasis, including endothelial activation and pro-inflammatory remodeling [49,51].

Mechanistically, reduced NO bioavailability and impaired vascular responsiveness to NO are central features of diabetic vasculopathy. Beyond reduced NO synthesis, “vascular NO resistance” reflects impaired downstream signaling and reduced vasodilatory responsiveness [52]. Hyperglycemia-induced reactive oxygen species generation, advanced glycation end-products, protein kinase C activation, and vascular insulin resistance collectively promote NO quenching and disrupt NO–cGMP signaling. These processes exacerbate endothelial activation, vascular inflammation, and arterial stiffening, thereby accelerating atherosclerotic progression and increasing cardiovascular risk [49,52]. Given the strong dependence of female vascular protection on intact NO signaling, diabetes-related disruption of NO pathways may represent a plausible biological mechanism underlying the disproportionate increase in cardiovascular risk observed in women with T2DM.

Importantly, microvascular dysfunction may act as a sex-relevant amplifier of cardiovascular risk in T2DM that is insufficiently captured by conventional clinical risk markers. Coronary microvascular dysfunction (CMD) is increasingly recognized as a mechanistic substrate for ischemia in the absence of obstructive epicardial coronary disease and as a contributor to adverse outcomes, including heart failure with preserved ejection fraction (HFpEF), a condition that disproportionately affects women [53,54]. Emerging evidence suggests that CMD exhibits sex-specific correlates and may reflect distinct pathophysiological endotypes in women compared with men [53]. In sex-stratified imaging cohorts, impaired myocardial perfusion reserve has been linked to adverse myocardial remodeling and functional vulnerability in women, independent of obstructive coronary disease [53].

Complementing these observations, prospective cohort data indicate that systemic microvascular endothelial dysfunction may have heightened clinical relevance for female-predominant heart failure pathways in diabetes. Microvascular endothelial dysfunction has been associated with increased risk of HFpEF in women with T2DM, but not in men [54].

From a life-course and prevention perspective, these vascular phenotypes are clinically important because endothelial and microvascular dysfunction often precede overt macrovascular events and may represent actionable intermediate targets for early intervention. Population-based data further demonstrate that both microvascular and macrovascular complications remain common in women with diabetes, underscoring the need for sex-aware risk stratification and targeted preventive strategies from the time of T2DM diagnosis [55].

Collectively, impaired NO signaling, endothelial activation, and microvascular dysfunction emerge as central mechanisms amplifying cardiovascular risk in women with T2DM—pathways that are not fully captured by traditional risk factor surveillance alone (Figure 1) [49,50,51,52,53,54,55].

From a nutritional perspective, these vascular phenotypes are highly sensitive to dietary composition and postprandial metabolic stress. Diets rich in polyphenol-containing plant foods (e.g., fruits, vegetables, tea, cocoa, and olive oil) have been shown to improve endothelial function and enhance nitric oxide bioavailability, partly through reductions in oxidative stress and inflammation [11,12]. In contrast, dietary patterns characterized by high intake of saturated fats and energy-dense, refined carbohydrate–rich foods may impair endothelial function and promote vascular inflammation by increasing oxidative stress and metabolic burden [42,43,46].

Postprandial dyslipidemia—exacerbated by refined carbohydrate-rich and energy-dense dietary patterns—further contributes to endothelial dysfunction through prolonged exposure to atherogenic remnant lipoproteins and oxidative stress [42,43]. Given that female vascular protection is more strongly dependent on intact nitric oxide signaling, these diet-induced perturbations may have disproportionate clinical consequences in women with T2DM [51].

Taken together, these observations support the concept that endothelial and microvascular dysfunction are not only central features of diabetic vascular disease but also key targets for nutrition-based, sex-specific cardiovascular prevention strategies.

## 5. Dietary Patterns and Cardiovascular Risk in Women with T2DM

### 5.1. Sex Differences in Dietary Patterns and Nutrient Intake in Type 2 Diabetes

Dietary patterns are a cornerstone of cardiovascular risk management in women with T2DM, with established effects on key cardiometabolic and vascular pathways. In T2DM populations, current evidence suggests that dietary behaviors and nutrient intake profiles differ between women and men, and that these differences may contribute to adverse postprandial metabolism, endothelial dysfunction, and increased cardiovascular risk.

Observational studies in adults with T2DM suggest that women generally report higher overall diet quality compared with men, including greater intakes of fruits, vegetables, and dietary fiber, alongside lower consumption of alcohol and red or processed meats. However, despite these seemingly favorable patterns, women with T2DM do not experience proportional cardiovascular protection, indicating that diet quality scores alone may inadequately capture sex-specific nutritional risk in diabetes [27,41]. This discrepancy highlights the importance of examining qualitative aspects of nutrient intake and metabolic context rather than relying solely on composite dietary indices.

In T2DM, sex differences in macronutrient metabolism may modify the cardiometabolic effects of habitual dietary intake. Women with T2DM often exhibit greater disturbances in postprandial glucose and lipid metabolism relative to metabolically healthy women, suggesting a loss of physiological flexibility in substrate handling. Diets characterized by a high proportion of refined carbohydrates may exert disproportionately adverse effects on postprandial glycemia and lipidaemia in women with T2DM, even when total carbohydrate intake is not excessive [42,43]. Conversely, inadequate dietary protein intake—reported more frequently among older women with T2DM—may contribute to loss of lean mass, reduced insulin sensitivity, and diminished metabolic resilience, with potential downstream effects on cardiovascular risk [56].

Micronutrient inadequacies may further impair insulin signaling, endothelial function, and inflammatory regulation in women with T2DM. Studies in diabetic cohorts indicate that women are more likely than men to exhibit suboptimal intakes or circulating levels of vitamin D, magnesium, and iron—micronutrients implicated in insulin action, endothelial function, and inflammatory regulation [57,58]. Such inadequacies may be exacerbated by energy-restricted diets, polypharmacy, and diabetes-related dietary avoidance patterns, particularly during menopause and older age [57,58].

Behavioral and psychosocial factors further shape dietary patterns in women with T2DM. Women are more likely to report restrictive dieting behaviors, weight cycling, and emotional eating, which may undermine long-term adherence to cardioprotective dietary recommendations and contribute to glycemic variability and metabolic instability. Additionally, evidence suggests that nutritional counseling in T2DM often lacks sex-specific tailoring, with dietary advice frequently extrapolated from male-dominated clinical trials and insufficiently adapted to female-specific metabolic and life-course contexts [41,59]. Sex-related differences in dietary behaviors and nutritional vulnerabilities in individuals with T2DM are summarized in Table 2.

Overall, data from T2DM-specific studies indicate that sex differences in dietary patterns extend beyond total energy intake or guideline adherence. Interactions between nutrient composition, micronutrient adequacy, and female-specific metabolic phenotypes may influence cardiovascular risk in women with T2DM. These findings support the need for sex-aware nutritional assessment and individualized dietary strategies as integral components of cardiovascular risk reduction in diabetic women.

### 5.2. Cardioprotective Dietary Patterns and Their Sex-Specific Implications in Women with T2DM

Dietary pattern-based approaches represent a cornerstone of cardiovascular risk reduction in T2DM. Beyond single nutrients, the overall dietary pattern more accurately captures the combined effects of food matrices and nutrient interactions on glycemic regulation, atherogenic dyslipidemia, blood pressure, systemic inflammation, and endothelial function—pathways that converge to determine long-term cardiovascular outcomes. In women with T2DM, who experience a disproportionate relative increase in cardiovascular risk, the relevance of cardioprotective patterns is shaped by sex-specific cardiometabolic phenotypes discussed earlier in this review, including adipose tissue dysfunction with ectopic fat susceptibility, impaired postprandial lipid handling, and microvascular and endothelial vulnerability.

**Mediterranean-style dietary patterns** are supported by the strongest evidence base for cardiovascular prevention in high-risk populations (Table 3).

In the PREDIMED trial, Mediterranean diet interventions supplemented with extra-virgin olive oil or nuts reduced major cardiovascular events compared with a control dietary advice group, with indirect relevance to populations with T2DM, given the high-cardiometabolic-risk profile of participants and the substantial proportion with diabetes [60]. In T2DM-focused evidence syntheses, Mediterranean-style diets are consistently associated with improvements in HbA1c, insulin sensitivity, triglycerides, and inflammatory markers, supporting their role as a default cardioprotective pattern when culturally feasible [12]. Mechanistically, high intakes of unsaturated fats (particularly from olive oil and nuts), dietary fiber, and polyphenol-rich plant foods have been shown to improve endothelial nitric oxide bioavailability, reduce oxidative stress, and attenuate low-grade inflammation—pathways especially pertinent in women, whose vascular protection is more dependent on intact endothelial function and who may exhibit greater adiposity-related inflammatory burden at diagnosis [61].

**DASH-style dietary patterns** offer complementary cardioprotective mechanisms, with a particular emphasis on blood pressure control—one of the most powerful modifiable determinants of cardiovascular outcomes in diabetes (Table 3). DASH patterns increase the intake of potassium- and magnesium-rich plant foods and low-fat dairy while reducing sodium and ultra-processed foods, thereby improving vascular function and blood pressure regulation. In T2DM populations, systematic review evidence supports the favorable effects of DASH dietary patterns on cardiometabolic outcomes [62]. Given that real-world cohorts frequently demonstrate suboptimal blood pressure target attainment in women with T2DM, dietary strategies that directly address sodium balance and vascular tone are particularly relevant for reducing female cardiovascular vulnerability.

**Carbohydrate-modified dietary strategies**, including low- and very-low-carbohydrate approaches, have gained attention primarily for glycemic management (Table 3). Meta-analytic evidence indicates that carbohydrate restriction can improve HbA1c, reduce body weight, and lower triglycerides—effects that may be clinically meaningful in the short to medium term [63]. However, the cardiovascular implications depend strongly on the quality of fat and protein sources replacing carbohydrates. Evidence syntheses comparing dietary approaches for weight management in T2DM suggest that long-term superiority of any single macronutrient distribution is uncertain and that adherence and overall dietary quality are critical determinants of sustained benefit [64]. This nuance is particularly important in women with T2DM, given sex differences in postprandial lipidaemia and ectopic fat deposition: low-carbohydrate patterns that increase saturated fat intake may worsen lipotoxic pathways, whereas carbohydrate reduction embedded within a Mediterranean- or plant-forward, unsaturated-fat-rich pattern may better align with cardiovascular risk reduction.

**Plant-based dietary patterns** (plant-forward, vegetarian, or vegan variants) are also associated with favorable cardiometabolic profiles (Table 3). Evidence syntheses link plant-based patterns to improved glycemic control, body weight, and lipid parameters, largely mediated by higher dietary fiber intake, lower energy density, and improved fat quality [12,65]. These mechanisms map directly onto sex-specific phenotypes relevant to diabetic women, including adipose tissue inflammation, ectopic fat susceptibility, and endothelial dysfunction. Nevertheless, direct evidence on sex-stratified dietary responsiveness remains limited across pattern trials, underscoring the need for designs that incorporate female-relevant intermediate vascular endpoints (e.g., endothelial function, arterial stiffness, microvascular dysfunction) in addition to standard glycemic and lipid outcomes [12,65].

**Table 3:** Cardioprotective dietary patterns and their sex-specific relevance for cardiovascular risk reduction in women with type 2 diabetes.

Dietary Pattern	Key Nutritional Features	Cardiometabolic Effects in Individuals with T2DM	Sex-Specific Relevance in Women with T2DM	References
**Mediterranean Diet**	High intake of vegetables, fruits, legumes, whole grains, nuts, olive oil, and fish; low intake of red and processed meat; rich in monounsaturated fats, fiber, and polyphenols	Improves glycemic control, insulin sensitivity, postprandial lipid metabolism, and inflammatory profiles; associated with reduced cardiovascular events in high-risk populations	Particularly relevant in women with T2DM by improving endothelial function, reducing oxidative stress and adipose tissue inflammation, and helping counterbalance loss of vascular protection across midlife transitions	Kahleova et al. [12]; Estruch et al. [60]
**DASH dietary pattern**	High intake of fruits, vegetables, whole grains, legumes, and low-fat dairy; reduced sodium intake; low intake of ultra-processed foods and red meat; rich in potassium, magnesium, and calcium	Improves blood pressure regulation, vascular function, and overall cardiometabolic risk profile in T2DM	Particularly relevant in women with T2DM, in whom suboptimal blood pressure control contributes to excess cardiovascular risk; supports endothelial function through improved micronutrient balance	Stedman et al. [21]; Chiavaroli et al. [62]
**Plant-based/plant-forward dietary patterns**	Predominantly plant-derived foods, including vegetables, fruits, legumes, whole grains, nuts, and seeds; reduced intake of animal-source foods; high fiber and low energy density	Improves glycemic control, body weight, lipid profile, and inflammatory status	Particularly beneficial in women with T2DM by reducing adipose tissue inflammation, limiting ectopic fat deposition, and improving endothelial function	Kahleova et al. [12]; Qian et al. [65]
**Low-carbohydrate dietary approaches**	Reduced carbohydrate intake with replacement by fat and/or protein; metabolic effects depend on quality of replacement foods	Reduces HbA1c, body weight, and triglyceride concentrations, particularly in the short to medium term	Cardiovascular effects depend strongly on fat quality; in women with T2DM, saturated-fat-rich patterns may exacerbate lipotoxicity and ectopic fat accumulation, whereas unsaturated-fat-based approaches are more favorable	Goldenberg et al. [63]; Churuangsuk et al. [64]
**Unsaturated-fat-rich carbohydrate-modified patterns**	Moderate carbohydrate restriction combined with higher intake of unsaturated fats from olive oil, nuts, seeds, and fish	Improves metabolic flexibility, insulin sensitivity, and lipid profile while reducing triglycerides	Particularly well aligned with sex-specific metabolic phenotypes in women with T2DM, including susceptibility to ectopic fat deposition, lipotoxicity, and endothelial dysfunction	Kahleova et al. [12]; Schwingshackl et al. [47]; Goldenberg et al. [63]

Abbreviations: DASH, Dietary Approaches to Stop Hypertension; HbA1c, glycated hemoglobin; T2DM, type 2 diabetes mellitus.

Key shared mechanisms include improved insulin sensitivity and endothelial function. Importantly, hormonally sensitive transitions—especially the menopause transition—are associated with adverse shifts in adipose tissue distribution, blood pressure, lipoprotein profiles, and vascular function, amplifying cardiovascular vulnerability of sustained cardioprotective dietary adherence in midlife and older women with T2DM [66].

Within the diet or genes or both framework, cardioprotective patterns also represent key platforms for precision nutrition: inter-individual variability in response (e.g., lipid and glycemic responses to fat quality or carbohydrate load) likely reflects, at least in part, genetic susceptibility and epigenetic regulation, while long-term adherence to high-quality dietary patterns has been linked to cardiometabolic risk modulation in integrative evidence syntheses [61]. However, robust sex-stratified evidence on diet–gene or diet–epigenetic interactions in T2DM remains critically limited, representing a priority knowledge gap for future trials in women. Given the marked heterogeneity in metabolic and vascular phenotypes among women with T2DM, the potential sex-specific relevance of major cardioprotective dietary patterns should be considered in addition to their overall cardiometabolic benefits.

In summary, Mediterranean and DASH-style patterns, plant-forward dietary models, and carefully constructed carbohydrate-modified approaches can all improve cardiometabolic risk factors in T2DM. Yet, in women, baseline vascular vulnerability and life-course transitions likely modulate both absolute benefit and the most clinically relevant mechanisms. Future randomized studies with prespecified sex-stratified analyses and vascular intermediate endpoints are needed to enable truly sex-aware, precision dietary strategies for cardiovascular risk reduction in women with T2DM [12,60,61,62,63,64,65,66].

### 5.3. Pro-Inflammatory Diets and Advanced Glycation End Products

Pro-inflammatory dietary patterns represent a critical and modifiable contributor to cardiovascular risk in women with T2DM, in whom inflammatory and endothelial pathways are already dysregulated. Among these, diets characterized by high consumption of ultra-processed foods (UPFs) have gained increasing attention due to their consistent associations with obesity, insulin resistance, systemic inflammation, and adverse cardiovascular outcomes [67,68]. UPFs are typically energy-dense, low in dietary fiber and micronutrients, and enriched in refined carbohydrates, saturated fats, salt, and food additives—features that promote insulin resistance, adipose tissue inflammation, ectopic fat accumulation, and endothelial dysfunction [69,70].

Controlled feeding studies provide causal evidence that ultra-processed diets increase energy intake and weight gain, thereby exacerbating adiposity-related and inflammatory pathways that are particularly relevant in women with T2DM. In a landmark inpatient randomized trial, consumption of an ultra-processed diet led to increased ad libitum energy intake and rapid weight gain compared with a minimally processed diet, despite matched macronutrient composition [46]. Complementing these experimental findings, systematic reviews and meta-analyses consistently report associations with elevated inflammatory biomarkers, including C-reactive protein and interleukin-6, and with increased cardiovascular risk [39,67,68]. These inflammatory effects are mechanistically relevant in T2DM, a condition already characterized by chronic low-grade inflammation and heightened cardiometabolic vulnerability.

In women with T2DM, pro-inflammatory dietary exposures have particularly adverse vascular consequences. Women often develop diabetes at a higher level of adiposity and metabolic burden, and diabetes appears to abrogate the physiological female advantage in endothelial function and lipid handling [3,9,17]. Experimental and clinical evidence suggests that female vascular protection relies more heavily on intact endothelial NO signaling; thus, dietary factors that impair NO bioavailability and promote oxidative stress directly target a central pathway of female vascular protection [51,71]. During hormonally sensitive transitions such as menopause, when estrogen-mediated vascular protection is lost, susceptibility to diet-induced inflammation and endothelial dysfunction is further amplified [20,66].

Advanced glycation end products (AGEs) constitute an additional dietary pathway linking pro-inflammatory diets to vascular dysfunction in diabetes. Advanced AGEs are generated endogenously under hyperglycemic conditions and are also ingested through highly processed and heat-treated foods [49,50]. Dietary AGE intake has been shown to increase oxidative stress, activate inflammatory signaling via the receptor for AGEs, and impair endothelial NO-dependent vasodilation—key mechanisms in the development of diabetic vasculopathy [52,72].

Importantly, recent clinical and translational studies indicate that dietary AGEs can directly impair vascular endothelial function in individuals with T2DM. Evidence from intervention and mechanistic studies demonstrates that high dietary AGE exposure worsens markers of endothelial dysfunction and insulin resistance, whereas AGE-restricted diets improve oxidative stress profiles and vascular function [73,74,75]. These mechanisms converge with the concept of vascular NO resistance in T2DM, characterized by impaired downstream responsiveness to NO despite preserved or compensatory NO production [52]. Given the central role of NO signaling in female vascular health, AGE-mediated disruption of endothelial pathways represent a sex-relevant mechanism contributing to the disproportionate cardiovascular risk observed in women with diabetes [51,72,75].

Ultra-processed and AGE-rich dietary patterns act as potent metabolic and vascular stressors that accelerate endothelial dysfunction and cardiometabolic deterioration in T2DM.

These dietary exposures provide a key interface through which nutritional factors interact with genetic susceptibility and epigenetic regulation to shape cardiovascular risk in women with T2DM. Inter-individual variability in inflammatory responsiveness, AGE detoxification capacity, and endothelial resilience is influenced by both genetic susceptibility and sex-specific biological factors. Thus, pro-inflammatory diets and dietary AGEs provide a biologically plausible interface through which nutritional exposures may interact with genetic and epigenetic mechanisms to shape cardiovascular risk in women with T2DM, setting the stage for diet–gene and diet–epigenetic interactions discussed in the following section.

## 6. Diet–Gene and Diet–Epigenetic Interactions in Diabetic Women

### 6.1. Nutrigenetic Variants Relevant to Cardiometabolic Risk

**Figure 2:**
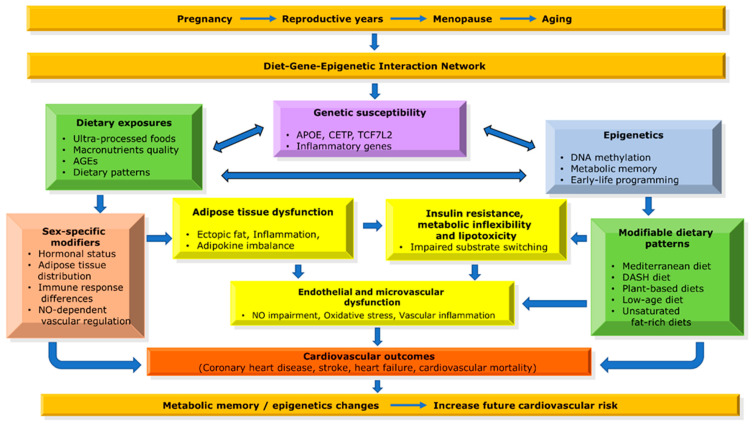
Integrated model of diet–gene–epigenetic interactions in cardiovascular risk in women with type 2 diabetes mellitus.

Women with T2DM exhibit substantial heterogeneity in metabolic responses to dietary exposures, driven in part by genetic susceptibility and sex-specific biological mechanisms [12]. Nutrigenetic studies demonstrate that common genetic variants influencing lipid metabolism, insulin signaling, and inflammatory pathways can modify metabolic and vascular responses to diet, thereby shaping long-term cardiometabolic trajectories [15,76]. Importantly, growing evidence indicates that effect sizes differ between women and men, although this remains insufficiently characterized due to limited sex-disaggregated data [9,19]. These sex-modified effect sizes are particularly relevant in diabetic women, in whom the loss of physiological vascular protection unmasks genetic susceptibility to diet-induced dyslipidemia, insulin resistance, and inflammation, ultimately contributing to disproportionate cardiovascular risk [9,17,19]. Key nutrigenetic interactions relevant to cardiometabolic risk in women with T2DM are summarized in Figure 2.

From a nutritional perspective, gene–diet interactions in T2DM are strongly influenced not only by overall dietary patterns but also by specific nutrient classes and food sources that modulate lipid metabolism, insulin signaling, and vascular function. In particular, dietary fat quality, carbohydrate quality, and intake of bioactive plant-derived compounds play a central role in shaping the phenotypic expression of genetic variants. Diets rich in monounsaturated and polyunsaturated fatty acids from olive oil, nuts, seeds, and fish are associated with more favorable lipid and metabolic responses, whereas saturated-fat-rich and refined-carbohydrate-rich dietary patterns may amplify genetic susceptibility to dyslipidemia and insulin resistance [11,12,47]. Similarly, carbohydrate quality—particularly dietary fiber content, glycemic index/load, and degree of processing—modifies glycemic responses in genetically susceptible individuals, reinforcing the importance of food composition in nutrigenetic risk modulation [42,43].

#### 6.1.1. Genetic Variants in Lipid Metabolism

Genes involved in lipid transport and lipoprotein remodeling represent a central axis through which diet interacts with cardiovascular risk in T2DM. Among these, apolipoprotein E (APOE) polymorphisms have been most extensively studied. The ε4 allele is consistently associated with higher low-density lipoprotein cholesterol (LDL-C) levels and increased cardiovascular risk, whereas the ε2 allele is linked to altered triglyceride-rich lipoprotein metabolism [42,76].

Importantly, dietary fat quality modifies the phenotypic expression of APOE variants. Diets rich in saturated fatty acids tend to amplify adverse lipid responses in ε4 carriers, whereas unsaturated-fat-rich dietary patterns—particularly those emphasizing monounsaturated and polyunsaturated fatty acids from olive oil, nuts, seeds, and fish—attenuate LDL-C elevations and improve overall lipoprotein profiles [76,77]. This distinction highlights that cardiovascular risk associated with APOE genotype is not determined solely by genetic background, but is strongly modulated by the qualitative composition of dietary fat its food sources. Although many nutrigenetic studies have not been powered for sex-stratified analyses, emerging evidence suggests that women may exhibit greater lipid sensitivity to dietary fat quality in the context of APOE genotype, although sex-stratified evidence remains limited [9,19].

Variants in the cholesteryl ester transfer protein (CETP) gene further illustrate sex-dependent nutrigenetic interactions. CETP polymorphisms influence high-density lipoprotein (HDL) metabolism and cholesterol transport, with dietary fat composition modulating these effects in a sex-dependent manner [78]. Contemporary observational and intervention studies indicate that CETP variants may differentially affect HDL-C responses to dietary fat intake in women compared with men, potentially reflecting sex-specific differences in HDL metabolism, reverse cholesterol transport, and hormonal regulation of lipid fluxes [17,78]. In women with T2DM—who often exhibit a more atherogenic lipoprotein phenotype despite similar or higher HDL-C levels—such gene–diet interactions may have disproportionate implications for residual cardiovascular risk. From a clinical nutrition perspective, these findings suggest that women with T2DM carrying lipid-related risk variants may derive greater benefit from dietary patterns emphasizing unsaturated fats and minimally processed food sources rather than from low-fat strategies that do not adequately address fat quality [12,47,78].

#### 6.1.2. Genetic Determinants of Insulin Signaling and Glucose Metabolism

Genetic variants affecting insulin secretion and insulin sensitivity represent another important layer of diet–gene interaction in T2DM. Polymorphisms in genes involved in insulin signaling and β-cell function—including transcriptional regulators of glucose homeostasis such as *TCF7L2*—have been shown to modify glycemic responses to dietary exposures and lifestyle interventions, contributing to clinically meaningful inter-individual heterogeneity in fasting glucose and related glycemic traits [79]. More broadly, genome-wide approaches indicate that macronutrient composition can interact with genetic background to influence glycemic phenotypes, supporting the concept that diet and inherited susceptibility jointly shape glucose metabolism beyond the effects of diet alone [80]. Consistent with this framework, contemporary reviews emphasize that carbohydrate quantity and, critically, carbohydrate quality (e.g., dietary fiber content, glycemic index/load, degree of processing) can differentially affect glycemia in genetically susceptible individuals; high-glycemic-load or -refined-carbohydrate patterns may exacerbate hyperglycemia and insulin resistance, whereas high-fiber, low-glycemic-index dietary patterns can attenuate postprandial excursions and improve overall glycemic control [81,82].

Sex-specific modulation of these associations is increasingly recognized. Women generally demonstrate greater insulin sensitivity than men prior to the development of overt dysglycemia; however, this advantage diminishes across hormonally and metabolically vulnerable states (notably menopause and established T2DM), resulting in a convergence toward similar—or in some contexts worse—insulin resistance profiles in women with diabetes [83]. Genetic susceptibility to impaired insulin signaling may therefore become clinically manifest at a later, but metabolically more advanced, stage in women, potentially magnifying diet-induced dysglycemia and downstream cardiovascular risk.

These observations support a nutrition-specific interpretation of gene–diet interactions, in which carbohydrate quality—not merely total carbohydrate quantity—plays a central role. High-fiber, low-glycemic-index, and minimally processed carbohydrate sources may attenuate genetically mediated dysglycemia, whereas refined and high-glycemic-load dietary patterns may exacerbate metabolic risk in susceptible women with T2DM.

#### 6.1.3. Inflammation-Related Polymorphisms and Diet-Induced Vascular Risk

Chronic low-grade inflammation is a defining feature of T2DM and a key mechanistic driver of endothelial dysfunction, atherosclerosis, and cardiovascular disease [22,49]. Genetic variation in inflammatory and immune-regulatory pathways may therefore modify individual susceptibility to diet-induced metabolic and vascular injury [84]. Polymorphisms affecting cytokine signaling, innate immune activation, and immune checkpoint regulation have been shown to influence inflammatory tone and cardiometabolic risk, particularly in the context of obesogenic and pro-inflammatory dietary patterns [39,85,86].

While most nutrigenetic research in diabetes has traditionally focused on lipid and glucose metabolism, emerging evidence supports a broader framework in which immune-regulatory genetic variants interact with environmental exposures—including diet—to shape metabolic phenotypes. In this context, polymorphisms in immune checkpoint and transcriptional regulatory genes such as CTLA4, FOXO3, and PTPN22 have been shown to interact with environmental factors to influence disease expression in diabetes, particularly in immune-mediated forms of the disease (type 1 diabetes) [87]. Although this evidence derives primarily from type 1 diabetes, it provides important mechanistic insight into how immune–genetic interactions may extend beyond classical metabolic pathways and could also be relevant to cardiometabolic risk through shared inflammatory and endothelial processes.

This immune–metabolic interface is particularly relevant for cardiovascular risk stratification, as inflammatory activation amplifies endothelial dysfunction, oxidative stress, and microvascular damage—processes that are already heightened in T2DM [49,88]. Pro-inflammatory dietary exposures, including ultra-processed foods and AGEs, have been consistently associated with elevated systemic inflammatory markers and impaired vascular function, and may further exacerbate these pathways in genetically susceptible individuals [72,86]. Importantly, sex-specific differences in immune and inflammatory signaling are increasingly recognized, with women exhibiting distinct immune response profiles and a greater reliance on intact endothelial nitric oxide signaling for vascular protection [19,51]. Once diabetes-related metabolic stress is established, unfavorable combinations of pro-inflammatory dietary patterns and immune-related risk genotypes may therefore have disproportionate cardiovascular consequences in women.

Collectively, these observations support the inclusion of immune-regulatory polymorphisms within a comprehensive nutrigenetic framework of diabetic cardiovascular risk. Although direct evidence in women with T2DM remains limited, available data suggest that genetic susceptibility to inflammation may be significantly modulated by dietary exposures. In particular, pro-inflammatory dietary patterns—characterized by ultra-processed foods, saturated-fat-rich foods, and refined carbohydrate intake—may amplify genetically mediated inflammatory and vascular responses, whereas minimally processed, fiber-rich, plant-forward dietary patterns may help attenuate these pathways.

#### 6.1.4. Sex-Modified Effect Sizes: Implications for Cardiometabolic Risk

A critical limitation of the current nutrigenetic literature is the relative scarcity of adequately powered sex-stratified analyses and consistent sex-disaggregated reporting, which can mask clinically relevant genotype effects when direction or magnitude differs between women and men [89,90]. Nevertheless, converging lines of evidence indicate that women and men differ not only in baseline cardiometabolic phenotypes but also in biological susceptibility to diet-related metabolic perturbations, supporting the plausibility of sex-modified effect sizes in gene–diet associations [9,90]. In women with T2DM, gene–diet interactions are likely to become clinically relevant during hormonally sensitive transitions, particularly menopause [20]. These changes likely narrow—or eliminate—the pre-existing female advantage in metabolic homeostasis and amplify the phenotypic expression of genetic variants affecting lipid handling, insulin signaling, or inflammatory regulation under suboptimal dietary exposures [9,19,20].

Importantly, emerging genetic evidence supports the concept that sex and sex hormones can modify diabetes-related genetic architecture and risk associations, providing mechanistic grounding for sex-aware nutrigenetic models [91]. Complementary experimental evidence also demonstrates sex-dependent cardiometabolic responses to obesogenic diets and downstream myocardial/vascular remodeling pathways, reinforcing biological plausibility for amplified genetic effects in women during vulnerable life-course windows [17].

Taken together, nutrigenetic variants influencing lipid metabolism, insulin signaling, and inflammation provide a biologically plausible framework through which diet and genetic susceptibility jointly shape cardiometabolic risk in diabetic women. Recognition of sex-modified effect sizes is essential for translating nutrigenetic insights into clinically meaningful, sex-specific dietary strategies aimed at reducing cardiovascular risk in T2DM [9,89,90].

From a nutritional perspective, these findings support the importance of dietary quality—particularly fat quality, carbohydrate quality, and degree of food processing—as key modifiable determinants of genetically mediated cardiometabolic risk. Dietary patterns emphasizing unsaturated fats, fiber-rich and minimally processed carbohydrate sources, and plant-based foods may help attenuate the phenotypic expression of risk alleles, whereas diets high in saturated fats and refined, ultra-processed foods may amplify genetic susceptibility.

These considerations underscore the need for sex-aware, nutrition-focused precision strategies that integrate genetic background with dietary composition, and that aim to reduce cardiovascular risk in women with T2DM through targeted modulation of diet–gene interactions across the life course.

### 6.2. Epigenetic Programming Across the Female Life Course

Epigenetic mechanisms represent a key interface through which diet and metabolic stress exert long-term effects on cardiovascular risk in women with T2DM. In the context of T2DM, epigenetic programming has emerged as a key contributor to long-term cardiometabolic risk, offering mechanistic insight into how early-life conditions, chronic hyperglycemia, and dietary patterns interact with genetic susceptibility across the female life course [16,92]. Importantly, epigenetic regulation is highly sensitive during specific developmental and hormonal windows, many of which are unique or particularly consequential in women.

From a nutritional perspective, these epigenetic processes are strongly influenced by dietary composition, including the intake of methyl-donor nutrients, fatty acid quality, and bioactive compounds such as polyphenols, which together link habitual diet to long-term vascular and metabolic risk.

#### 6.2.1. Intrauterine Environment and Early-Life Programming

The intrauterine environment represents a critical window for epigenetic programming with lifelong consequences for metabolic and cardiovascular health. Maternal hyperglycemia, gestational diabetes mellitus (GDM), and suboptimal maternal nutrition have been consistently associated with altered DNA methylation patterns in offspring genes involved in glucose metabolism, insulin signaling, adipogenesis, and inflammatory regulation [18,93,94]. These epigenetic alterations are detectable at birth and may persist into childhood and adulthood, predisposing offspring—particularly daughters—to insulin resistance, obesity, and cardiometabolic disease later in life [93,94].

Evidence from human cohort studies demonstrates that exposure to maternal diabetes or hyperglycemia in utero is associated with differential methylation at loci regulating β-cell function, lipid metabolism, and vascular pathways, supporting a mechanistic link between prenatal metabolic stress and future cardiometabolic vulnerability [18,95]. Given the strong influence of maternal metabolic status on the intrauterine milieu, women with diabetes or GDM represent a key population in whom nutritional and glycemic optimization during pregnancy may have intergenerational benefits.

#### 6.2.2. Hyperglycemia, Diet, and DNA Methylation in T2DM

Beyond early development, chronic hyperglycemia and dietary exposures in adulthood can actively reshape the epigenome, contributing to the phenomenon of “metabolic memory” observed in diabetes. Persistent hyperglycemia has been shown to induce stable DNA methylation changes in genes regulating oxidative stress, inflammation, endothelial function, and insulin signaling—pathways central to diabetic vascular complications [92,96]. These epigenetic marks may persist even after glycemic improvement, potentially explaining the long-lasting cardiovascular risk observed in individuals with a history of poor metabolic control.

Dietary patterns act as powerful modulators of epigenetic regulation through multiple mechanisms, including the availability of methyl donors, modulation of one-carbon metabolism, and regulation of histone-modifying enzymes. Emerging human evidence indicates that dietary quality—particularly adherence to fiber-rich, plant-forward, and polyphenol-containing dietary patterns—is associated with favorable DNA methylation signatures linked to lower cardiometabolic risk and reduced inflammation [16,97]. Conversely, diets rich in ultra-processed foods, saturated fats, and advanced glycation end products may promote adverse epigenetic remodeling, amplifying inflammatory and endothelial dysfunction pathways relevant to cardiovascular disease in T2DM [72]. In contrast, diets emphasizing vegetables, fruits, whole grains, legumes, olive oil, and other polyphenol-rich plant foods may support more favorable epigenetic profiles through effects on oxidative stress, inflammation, and metabolic regulation [16,97].

#### 6.2.3. Transgenerational Risk and Female-Specific Implications

Epigenetic programming provides a biologically plausible mechanism for the transgenerational transmission of cardiometabolic risk. Animal and human data suggest that epigenetic modifications induced by maternal hyperglycemia, obesity, or dietary imbalance can be transmitted across generations, influencing disease susceptibility in offspring independent of shared genetics [93,98]. This phenomenon may be particularly relevant in women, who serve as both recipients and transmitters of metabolic and epigenetic information across the life course.

From a sex-specific perspective, female offspring appear especially vulnerable to the long-term cardiometabolic consequences of adverse intrauterine and early-life exposures, potentially reflecting sex differences in placental biology, hormonal regulation, and epigenetic responsiveness [19,95]. Moreover, women experience multiple hormonally sensitive transitions—including puberty, pregnancy, and menopause—during which epigenetic regulation may be further remodeled in response to metabolic and dietary stress. These cumulative epigenetic effects may contribute to the disproportionate cardiovascular risk observed in women with T2DM, particularly later in life.

Collectively, these findings underscore the importance of viewing epigenetic programming as a dynamic, life-course process through which diet and metabolic health interact with genetic susceptibility to shape cardiovascular risk in women with diabetes. From a nutritional perspective, maternal dietary quality, glycemic control, and long-term adherence to minimally processed, micronutrient-adequate dietary patterns may influence not only individual vascular risk but also intergenerational cardiometabolic vulnerability.

These observations highlight the potential for dietary interventions targeting nutrient quality and food composition to modulate epigenetic pathways relevant to inflammation, endothelial function, and metabolic homeostasis. Integrating epigenetic perspectives into nutrigenetic and precision nutrition frameworks may enhance early risk stratification and support the development of targeted, sex-aware preventive strategies with potential benefits extending beyond a single generation.

### 6.3. Diet as an Epigenetic Modulator

Diet represents one of the most powerful and modifiable environmental determinants of epigenetic regulation, acting through multiple nutrient-sensitive pathways to influence DNA methylation, histone modifications, and non-coding RNA expression. In the context of T2DM, dietary modulation of the epigenome provides a mechanistic framework linking habitual nutritional exposures to long-term cardiometabolic and cardiovascular risk. Importantly, emerging evidence suggests that epigenetic responsiveness to diet may be sex-dependent and particularly relevant in women across hormonally sensitive life stages.

#### 6.3.1. Polyphenols and Epigenetic Regulation

Dietary polyphenols—abundant in fruits, vegetables, whole grains, tea, coffee, olive oil, and cocoa—have been extensively studied for their capacity to modulate epigenetic processes relevant to metabolic and vascular health. Experimental and translational human studies demonstrate that polyphenols can influence DNA methyltransferase activity, histone acetylation, and microRNA expression, thereby regulating genes involved in inflammation, oxidative stress, insulin signaling, and endothelial function [99,100]. These effects are particularly relevant in T2DM, where chronic low-grade inflammation and oxidative stress drive progressive vascular dysfunction.

Recent human intervention studies and integrative reviews indicate that adherence to polyphenol-rich dietary patterns, such as Mediterranean-style diets, is associated with favorable DNA methylation signatures linked to improved cardiometabolic profiles and reduced cardiovascular risk [100,101]. From a sex-specific perspective, polyphenol-mediated epigenetic effects may be especially salient in women, whose vascular protection relies more heavily on intact endothelial nitric oxide signaling—a pathway that is sensitive to both oxidative stress and epigenetic regulation. Although direct sex-stratified epigenetic data remain limited, these findings support a biologically plausible role for polyphenol-rich diets as epigenetic modulators of cardiovascular risk in women with T2DM.

#### 6.3.2. Fatty Acid Composition and Epigenetic Remodeling

Beyond total fat intake, the qualitative composition of dietary fatty acids exerts distinct epigenetic effects with implications for cardiometabolic risk. Unsaturated fatty acids, particularly omega-3, polyunsaturated fatty acids (PUFAs), have been shown to influence DNA methylation and histone modification patterns in genes regulating lipid metabolism, inflammation, and insulin sensitivity [102,103]. In contrast, diets enriched in saturated fatty acids may promote adverse epigenetic remodeling, reinforcing pro-inflammatory and insulin-resistant phenotypes.

Recent epigenome-wide association studies and dietary intervention trials suggest that fatty acid-dependent epigenetic changes may contribute to inter-individual variability in metabolic responses to diet, including differential effects on lipid profiles and inflammatory markers [103,104]. Given known sex differences in lipid handling and adipose tissue biology, epigenetic responses to fatty acid composition may differ between women and men, potentially amplifying cardiovascular vulnerability in women with T2DM when dietary fat quality is suboptimal. These observations reinforce the importance of emphasizing fat quality—not merely macronutrient distribution—in dietary strategies targeting epigenetically mediated cardiometabolic risk. These effects may be particularly relevant in women with T2DM, who exhibit sex-specific differences in lipid metabolism and adipose tissue biology.

#### 6.3.3. Methyl-Donor Nutrients: Folate, Vitamin B12, and One-Carbon Metabolism

Methyl-donor nutrients, including folate and vitamin B12, play a central role in one-carbon metabolism and are essential for the maintenance of normal DNA methylation patterns. Inadequate intake or impaired metabolism of these nutrients can disrupt methylation capacity, leading to aberrant gene expression in pathways relevant to glucose metabolism, inflammation, and vascular function [105,106]. In individuals with T2DM—particularly women, who may be more susceptible to micronutrient inadequacy due to dietary restriction, aging, or medication use—such disruptions may have clinically meaningful consequences.

Recent observational and mechanistic studies indicate that folate and vitamin B12 status is associated with global and locus-specific DNA methylation patterns linked to cardiometabolic risk factors, including insulin resistance and endothelial dysfunction [106,107]. Moreover, emerging evidence suggests that adequate methyl-donor availability may mitigate some of the adverse epigenetic effects of hyperglycemia and oxidative stress, highlighting a potential role for targeted nutritional optimization as part of precision prevention strategies in diabetes [108].

Collectively, these findings underscore diet’s capacity to act as an epigenetic modulator across multiple nutrient classes. Polyphenols, fatty acid composition, and methyl-donor nutrients converge on shared epigenetic pathways that regulate inflammation, metabolic flexibility, and vascular health. In women with T2DM—who experience cumulative metabolic stress across hormonally sensitive life stages—dietary modulation of the epigenome may represent a particularly promising avenue for reducing long-term cardiovascular risk and for complementing sex-aware nutrigenetic approaches.

## 7. Critical Windows Across the Female Life Course in Cardiovascular Risk Among Women with T2DM

Across the female life course, several biologically sensitive periods are characterized by metabolic, hormonal, and vascular remodeling that strongly influences long-term cardiovascular risk. During these periods, heightened cardiometabolic plasticity increases vulnerability to hyperglycemia, insulin resistance, inflammation, and adverse dietary exposures. In women with T2DM or its precursors, these periods represent not only phases of accelerated cardiovascular risk accumulation but also strategic opportunities for targeted nutritional and lifestyle interventions. Importantly, during these windows, diet–gene and diet–epigenetic interactions may exert disproportionate and durable effects on cardiovascular health [3,9,20].

### 7.1. Pregnancy and Gestational Metabolic Stress

Pregnancy is a unique metabolic stressor characterized by progressive insulin resistance, physiological hyperlipidemia, and low-grade inflammation. However, when these adaptive processes exceed metabolic capacity, GDM emerges as a pathological manifestation of impaired glucose and lipid handling. Increasingly, GDM is conceptualized as a “cardiovascular stress test” that unmasks latent metabolic and vascular vulnerability in women who are predisposed to future T2DM and CVD [18,109,110].

Women with prior GDM have a substantially increased long-term risk of T2DM and major cardiovascular outcomes, including hypertension, ischemic heart disease, stroke, and heart failure [18,111]. Importantly, vascular dysfunction and adverse cardiometabolic profiles are often detectable within years after pregnancy, suggesting that GDM initiates or accelerates pathogenic processes rather than merely serving as a risk marker [109,112].

Mechanistically, GDM is associated with persistent endothelial dysfunction, increased arterial stiffness, and chronic low-grade inflammation, even after glycemic normalization postpartum [110,113]. These alterations converge with emerging concepts of vascular nitric oxide resistance and microvascular impairment, pathways that are particularly relevant for female cardiovascular health. Dietary exposures during pregnancy—such as excessive intake of refined carbohydrates, saturated fats, and ultra-processed foods—can exacerbate these mechanisms by promoting postprandial hyperglycemia, lipotoxicity, and inflammatory activation [114].

Pregnancy represents an early life-course window in which diet–gene interactions may contribute to clinically relevant metabolic heterogeneity. Genetic variants influencing insulin secretion, lipid metabolism, and inflammatory responses can modulate metabolic adaptation to pregnancy and dietary exposures, predisposing certain women to GDM under otherwise moderate nutritional stress [115,116]. In parallel, intrauterine exposure to hyperglycemia and suboptimal maternal nutrition induces epigenetic modifications in offspring genes involved in glucose metabolism, adipogenesis, and vascular regulation, thereby propagating intergenerational cardiometabolic risk [117,118]. Thus, pregnancy is both a critical period for maternal cardiovascular risk stratification and a key opportunity for targeted nutritional interventions with long-term and transgenerational benefits.

### 7.2. Menopause and Loss of Hormonal Protection

The menopause transition is a major cardiometabolic inflection point in the female life course. Declining estrogen signaling promotes adverse changes in adipose distribution, insulin sensitivity, lipid metabolism, blood pressure regulation, and vascular function, thereby accelerating cardiovascular risk [20,119,120]. In women with T2DM, menopause often marks the convergence of cumulative metabolic stress and loss of hormonal vascular protection, resulting in disproportionate increases in cardiovascular morbidity and mortality.

A central mechanism of this transition is disruption of the estrogen–lipid–insulin axis. Estrogen plays a critical role in maintaining insulin sensitivity, favorable lipoprotein profiles, and endothelial nitric oxide bioavailability. Its decline is associated with increased visceral and ectopic fat deposition, worsening insulin resistance, elevated triglycerides, and a shift toward a more atherogenic lipoprotein phenotype [121,122]. These changes exacerbate pre-existing diabetic cardiometabolic abnormalities and amplify vascular vulnerability, particularly in the microcirculation.

Menopause modifies dietary responsiveness. Evidence suggests that postmenopausal women exhibit attenuated metabolic responses to dietary interventions compared with premenopausal women, potentially due to altered substrate partitioning, reduced metabolic flexibility, and changes in gut microbiota composition [123,124]. Consequently, dietary patterns that were previously tolerated may become less effective in counteracting cardiometabolic deterioration after menopause.

Genetic susceptibility further modulates these processes. Variants affecting lipid handling, insulin signaling, and inflammatory regulation may exert stronger phenotypic effects after menopause, when estrogen-mediated buffering of metabolic stress is lost [125]. These observations underscore menopause as a critical window for sex-specific precision nutrition, during which dietary quality may strongly influence cardiovascular trajectories in women with T2DM.

### 7.3. Aging, Sarcopenic Obesity, and Nutritional Vulnerability

With advancing age, women with T2DM experience sarcopenia, adiposity redistribution, chronic inflammation, and declining metabolic resilience. Sarcopenic obesity, characterized by reduced skeletal muscle mass and excess adiposity, is strongly associated with insulin resistance, frailty, and cardiovascular risk [126,127]. Women are disproportionately affected due to longer lifespan, lower baseline muscle mass, and cumulative hormonal and metabolic stress across earlier life stages.

Loss of skeletal muscle mass impairs glucose disposal, reduces metabolic flexibility, and promotes ectopic fat accumulation, thereby accelerating cardiometabolic dysfunction. In this context, protein needs increase with aging, yet observational data indicate that older women with diabetes frequently fail to meet recommended protein intakes, particularly when energy restriction or dietary avoidance patterns are present [56,128]. Inadequate protein intake contributes to sarcopenia, impaired metabolic resilience, and potentially reduced vascular repair capacity.

Chronic low-grade inflammation further amplifies cardiovascular risk in older diabetic women. Pro-inflammatory dietary patterns, micronutrient inadequacies, and impaired anabolic signaling converge to worsen endothelial dysfunction and arterial stiffness [129,130]. Importantly, aging-related epigenetic drift may interact with long-standing hyperglycemia and dietary exposures, reinforcing adverse gene expression patterns linked to vascular dysfunction and metabolic disease [131].

Together, aging and sarcopenic obesity represent a late-life critical window in which nutritional vulnerability becomes clinically decisive. Precision nutrition strategies emphasizing adequate protein intake, anti-inflammatory dietary patterns, and maintenance of muscle mass may therefore be essential components of cardiovascular risk mitigation in older women with T2DM.

## 8. Clinical Implications: Toward Sex-Specific Precision Nutrition in Women with T2DM

Despite advances in cardiometabolic prevention, dietary recommendations for T2DM remain largely based on population-level evidence and are often applied similarly across sexes. Biological sex, hormonal transitions, metabolic phenotypes, and genetic susceptibility all influence cardiometabolic responses to dietary exposures. Consequently, conventional uniform dietary strategies may inadequately capture the complexity of cardiovascular risk in women with T2DM [9,41,132]. Incorporating sex-specific biological determinants into nutritional management may improve the precision of cardiovascular prevention in women with T2DM.

### 8.1. Limitations of Uniform Dietary Recommendations

Current dietary guidelines for T2DM emphasize macronutrient balance, caloric control, and adherence to cardioprotective dietary patterns. Although these recommendations improve cardiometabolic outcomes at the population level, they do not fully account for sex-related differences in adipose tissue biology, insulin sensitivity, lipid metabolism, and vascular regulation [9,41]. Women with T2DM often present with more advanced metabolic impairment at diagnosis, which may influence both the magnitude and mechanisms of dietary benefit [9].

Moreover, inter-individual variability in metabolic responses to dietary interventions is substantial, even within well-controlled clinical trials. This heterogeneity reflects the combined influence of genetic background, epigenetic regulation, microbiome composition, and environmental exposures. Precision nutrition frameworks emphasize that effective dietary strategies should account for biological determinants such as sex, metabolic phenotype, and genetic susceptibility [133]. In women with T2DM, failure to incorporate sex-specific physiology and life-course transitions may contribute to persistent gaps in cardiovascular risk reduction despite guideline-based care.

### 8.2. Life-Course Timing of Nutritional Interventions

The effectiveness of nutritional interventions may vary substantially across the female life course. Critical windows—including pregnancy, the menopause transition, and older age—are characterized by profound metabolic and hormonal remodeling that modifies cardiometabolic risk and may alter dietary responsiveness [20].

Pregnancy represents an early opportunity for risk identification and prevention. As discussed above, GDM identifies women at increased long-term cardiometabolic and cardiovascular risk, making pregnancy an early opportunity for nutritional risk stratification.

GDM is increasingly recognized as a marker of long-term cardiometabolic vulnerability, with affected women experiencing significantly elevated risks of subsequent T2DM and CVD [18,109]. Nutritional optimization during pregnancy and postpartum, including improved dietary quality and reduced ultra-processed food intake, may influence both maternal and intergenerational cardiometabolic trajectories.

During the menopause transition, dietary strategies emphasizing anti-inflammatory food patterns, improved fat quality, and adequate micronutrient intake may help attenuate accelerated cardiometabolic deterioration. Declining estrogen levels are associated with increased visceral adiposity, worsening insulin resistance, and deterioration in vascular function, all of which may amplify the metabolic consequences of suboptimal dietary patterns [20,66]. During this stage, dietary strategies emphasizing anti-inflammatory food patterns, improved fat quality, and adequate micronutrient intake may be particularly relevant for mitigating accelerated cardiometabolic deterioration.

In older women with T2DM, nutritional priorities shift toward preserving muscle mass, preventing sarcopenic obesity, and maintaining metabolic resilience. Adequate protein intake, micronutrient sufficiency, and dietary patterns supporting muscle function become critical components of cardiometabolic risk management in this population [56,128].

### 8.3. Metabolic Phenotyping and Individualized Dietary Strategies

Beyond life-course considerations, metabolic heterogeneity in T2DM supports more individualized dietary strategies. Variability in adipose tissue distribution, insulin resistance severity, postprandial lipid metabolism, and inflammatory status can influence the metabolic impact of specific dietary components [44].

For example, individuals with pronounced insulin resistance and ectopic fat accumulation may benefit from dietary patterns that improve metabolic flexibility and reduce lipotoxicity, including Mediterranean-style or plant-forward dietary models emphasizing unsaturated fats and high dietary fiber intake. Conversely, patients with sarcopenic obesity or reduced metabolic reserve may require dietary approaches that prioritize adequate protein intake and preservation of lean body mass [44,56].

These phenotyping approaches align with cardiometabolic precision medicine frameworks that recognize distinct metabolic subtypes of T2DM [134]. Incorporating metabolic phenotyping into dietary counseling may therefore enhance the effectiveness of nutrition-based cardiovascular prevention strategies in women with T2DM.

### 8.4. Genetic Susceptibility and Nutrigenetic Considerations

Genetic variation adds an important layer of biological heterogeneity to dietary responses in T2DM. From a clinical perspective, nutrigenetic variation may help explain why women with similar dietary patterns exhibit different cardiometabolic responses [76,133]. Although many studies remain underpowered for sex-stratified analyses, emerging evidence suggests that gene–diet interactions may exhibit sex-specific effect sizes, potentially reflecting differences in hormonal regulation and metabolic substrate handling.

In women with T2DM, genetic susceptibility may become particularly relevant during hormonally sensitive stages such as menopause. For instance, variants influencing lipid metabolism or insulin signaling may exert stronger phenotypic effects once estrogen-mediated metabolic buffering declines [19,125]. Integrating genetic information into nutritional assessment may improve future risk stratification and dietary personalization in high-risk women with T2DM.

### 8.5. Implications for Clinical Dietetics and Cardiometabolic Prevention

Clinically, translating these concepts into practice requires moving beyond uniform dietary prescriptions toward individualized, sex-aware nutritional strategies. In clinical dietetics, this approach may involve integrating life-course history (e.g., GDM, menopause status), metabolic phenotyping, and emerging genetic insights into personalized dietary counseling. Such strategies may enhance patient adherence and improve the effectiveness of lifestyle interventions aimed at reducing cardiovascular risk [9,132].

At the population level, incorporating sex-specific considerations into prevention frameworks may help address the persistent excess cardiovascular risk observed in women with T2DM. Precision nutrition approaches that account for biological sex, metabolic phenotype, and genetic susceptibility may contribute to more effective long-term cardiovascular prevention in women with T2DM [135].

## 9. Knowledge Gaps and Future Directions in Sex-Specific Precision Nutrition for Women with T2DM

Despite increasing recognition of sex differences in diabetes outcomes, major knowledge gaps remain in understanding how diet, metabolic phenotypes, and genetic susceptibility jointly shape cardiovascular risk in women with T2DM. Addressing these gaps is essential for advancing precision nutrition strategies and improving cardiovascular prevention in women with T2DM [89,90,136,137].

A major limitation of the current evidence base is the scarcity of randomized controlled trials specifically designed to evaluate sex differences in dietary interventions. Although numerous dietary studies have examined cardiometabolic outcomes in individuals with T2DM, women are frequently underrepresented or analyses are not adequately powered to detect sex-specific effects [89,90]. As a result, sex-specific differences in dietary responsiveness may remain obscured in current evidence syntheses and recommendations. Future clinical trials should incorporate prespecified sex-stratified analyses and recruit sufficient numbers of women to enable robust evaluation of female-specific cardiometabolic endpoints, including endothelial function, arterial stiffness, and microvascular dysfunction [89,90].

A second major gap concerns the limited quality and clinical interpretability of nutrigenetic and nutrigenomic evidence in women with T2DM. Genetic variation modifies cardiometabolic responses to dietary exposures, but most nutrigenetic studies remain underpowered, heterogeneous, and insufficiently sex-specific [136,137]. This limitation is particularly relevant for women with T2DM, in whom gene–diet interactions may be influenced by hormonal status, adipose tissue biology, and immune–metabolic signaling. Future studies should combine genomic data with high-quality dietary phenotyping and prespecified sex-stratified analyses to clarify the clinical relevance of nutrigenetic interactions for cardiovascular prevention [136,137].

Another critical area requiring further investigation is the integration of life-course perspectives into cardiometabolic nutrition research. Although hormonally sensitive transitions such as pregnancy, menopause, and aging clearly influence cardiovascular risk, most nutritional studies in T2DM do not explicitly address these stages [20,108,138]. Longitudinal studies examining dietary patterns across the female life course, beginning in early adulthood and extending through reproductive transitions and aging, could provide valuable insights into critical windows during which nutritional interventions may exert the greatest impact [138].

Nutriepigenomics and systems biology may help clarify the molecular mechanisms linking diet, metabolic health, and cardiovascular disease in women with T2DM. Multi-omics approaches, including epigenomics, metabolomics, and microbiome profiling, may help identify molecular signatures that predict responsiveness to dietary interventions [139]. Combining multi-omics data with clinical phenotyping may improve identification of high-risk subgroups of women with T2DM and support more targeted nutritional strategies [139].

Finally, greater attention should be directed toward implementation research and translation of precision nutrition concepts into clinical practice. Even when evidence-based dietary interventions are available, real-world adherence remains a significant challenge. Sociocultural determinants, health literacy, access to dietetic services, and practical barriers related to delivery and scalability may all shape dietary adherence in women with diabetes [140,141]. Future research should evaluate how sex-aware dietary interventions can be effectively implemented within clinical care pathways and public health strategies [140,141].

Advancing sex-specific precision nutrition in women with T2DM will require coordinated efforts across sex-stratified trials, nutrigenetic research, life-course epidemiology, and multi-omics integration. Addressing these gaps may improve cardiovascular risk stratification and support more personalized dietary interventions for women with T2DM [89,90,136,137,138,139,140].

## 10. Conclusions

Women with T2DM exhibit a disproportionately greater increase in cardiovascular risk compared with men, driven by interacting metabolic, hormonal, genetic, and environmental factors across the female life course in women with T2DM. Conventional cardiovascular risk factors alone do not fully explain this disparity. Instead, sex-specific cardiometabolic phenotypes—including adipose tissue dysfunction, impaired metabolic flexibility, endothelial and microvascular dysfunction, and chronic low-grade inflammation—contribute to accelerated vascular disease progression in women with T2DM.

Diet is a key modifiable determinant of cardiometabolic health that interacts with genetic susceptibility and epigenetic regulation. Dietary patterns modulate central biological pathways relevant to cardiovascular risk in women with T2DM, including insulin resistance, lipid metabolism, endothelial function, and inflammation. In parallel, nutrigenetic and nutriepigenetic mechanisms contribute to inter-individual variability in metabolic responses to dietary exposures and may influence long-term cardiovascular trajectories.

Hormonally sensitive transitions—including pregnancy, menopause, and aging—represent critical windows during which metabolic stress and dietary exposures may have amplified and durable effects on cardiovascular risk. These life-course stages are characterized by dynamic interactions between metabolic phenotypes, nutritional factors, and gene–environment regulation, potentially contributing to the accumulation and persistence of cardiovascular vulnerability in women with T2DM.

Collectively, these findings support a shift toward sex-aware precision nutrition frameworks in the prevention and management of cardiovascular disease in women with T2DM. Integrating dietary quality with metabolic phenotyping, genetic susceptibility, and life-course context may improve cardiovascular risk stratification and enable more targeted and effective nutritional strategies.

Further progress in this field will depend on well-designed, sex-stratified clinical studies and integrative research approaches that link diet, genetic susceptibility, and cardiometabolic phenotypes across the female life course. Such advances are essential to refine precision prevention strategies and to reduce the disproportionate cardiovascular burden experienced by women with T2DM.

## Figures and Tables

**Table 1:** Sex differences in cardiovascular outcomes associated with type 2 diabetes mellitus.

Cardiovascular Outcome	Evidence from Epidemiological Studies	Sex-Specific Findings	References
**CHD**	Large meta-analyses of prospective cohort studies demonstrate a substantially higher relative risk of CHD associated with T2DM in women compared with men.	Women with T2DM exhibit a ~ 40–50% higher excess relative risk of CHD compared with men with T2DM.	Huebschmann et al. [3]; Peters et al. [7]
**Stroke**	Population-based cohort analyses and meta-analyses consistently report a stronger association between T2DM and stroke risk in women.	Women with T2DM exhibit a ~25–30% higher relative risk of stroke compared with men with T2DM.	Peters et al. [6]
**Cardiovascular mortality**	Observational cohorts and pooled analyses show a greater increase in cardiovascular mortality among women with T2DM compared with men.	Women with T2DM exhibit a loss of baseline female protection against cardiovascular mortality observed in the non-diabetic population.	de Ritter et al. [2]; Huebschmann et al. [3]
**Overall CVD events**	Meta-analyses including multiple vascular outcomes indicate that T2DM confers a greater proportional increase in cardiovascular risk among women.	Women with T2DM exhibit a greater relative increase in overall CVD risk compared with men with T2DM.	de Ritter et al. [2]; Huebschmann et al. [3]; Peters et al. [8]
**Risk factor control in clinical practice**	Real-world clinical databases demonstrate sex differences in attainment of treatment targets for cardiovascular risk factors.	Women with T2DM exhibit lower attainment of guideline-recommended lipid and blood pressure targets compared with men.	Ramírez-Morros et al. [4]; Stedman et al. [21]

Abbreviations: CHD, coronary heart disease; CVD, cardiovascular disease; T2DM, type 2 diabetes mellitus.

**Table 2:** Sex-specific differences in dietary patterns, nutrient intake, and nutritional vulnerabilities in individuals with type 2 diabetes mellitus.

Nutritional Domain	Typical Observations in Women with T2DM	Potential Cardiometabolic Implications	References
**Overall diet quality**	Women with T2DM generally report higher overall diet quality, including greater intake of fruits, vegetables, and dietary fiber, and lower alcohol consumption compared with men.	Despite more favorable diet quality scores, women with T2DM do not experience proportional reductions in cardiovascular risk, suggesting that conventional dietary indices may not fully capture sex-specific metabolic vulnerability.	Vitale et al. [14]; Peters et al. [27]; Mauvais-Jarvis et al. [41]
**Carbohydrate quality and postprandial metabolism**	Women with T2DM may exhibit greater disturbances in postprandial glucose and lipid metabolism, particularly in response to diets rich in refined carbohydrates.	Increased postprandial dyslipidemia and glycemic excursions contribute to endothelial dysfunction and atherosclerotic progression.	Borén et al. [42]; Tomlinson et al. [43]
**Protein intake**	Older women with T2DM frequently report lower protein intake, particularly during energy-restricted diets or weight-management interventions.	Inadequate protein intake contributes to loss of lean body mass, reduced insulin sensitivity, and sarcopenic obesity, increasing long-term cardiovascular risk.	Volkert et al. [56]
**Micronutrient status**	Women with T2DM are more likely to exhibit suboptimal intakes and circulating levels of vitamin D, magnesium, and iron.	Micronutrient inadequacies impair insulin signaling, endothelial function, and inflammatory regulation, thereby potentially exacerbating cardiometabolic risk.	Pittas et al. [57]; Barbagallo et al. [58]
**Eating behaviors and psychosocial factors**	Women with T2DM more frequently report restrictive dieting, weight cycling, and emotional eating behaviors.	These behaviors impair long-term adherence to cardioprotective dietary patterns and contribute to glycemic variability and metabolic instability.	Martins-Filho et al. [13]; Mauvais-Jarvis et al. [41]
**Nutritional counseling and guideline application**	Dietary recommendations for diabetes management are often not sex-specific and frequently extrapolated from mixed or male-dominated cohorts.	Lack of sex-tailored nutritional guidance likely limits the effectiveness of dietary interventions for cardiovascular risk reduction in women with T2DM.	Mauvais-Jarvis et al. [41]; Franz et al. [59]

Abbreviations: T2DM, type 2 diabetes mellitus.

## Data Availability

No new research data was created or analyzed in this study. Data sharing is not applicable to this article.

## Abbreviations

AGEs—Advanced glycation end products, APOE—apolipoprotein E, CETP—cholesteryl ester transfer protein, CHD—coronary heart disease, CMD—coronary microvascular dysfunction, CVD—cardiovascular disease, ED—endothelial dysfunction, GDM—gestational diabetes mellitus, HDL—high-density lipoprotein, HFpEF—heart failure with preserved ejection fraction, LDL-C—low-density lipoprotein cholesterol, NO—nitric oxide, PUFAs—polyunsaturated fatty acids, T2DM—type 2 diabetes mellitus, UPFs—ultra-processed foods.
